# An NLR-VOZ seesaw: from asymmetric selection to synthetic broad-spectrum resistance

**DOI:** 10.1007/s44154-026-00334-0

**Published:** 2026-07-21

**Authors:** Yachun Su, Shoujian Zang, Tingting Sun, Chuihuai You, Youxiong Que

**Affiliations:** 1 Key Laboratory of Sugarcane Biology and Genetic Breeding, Ministry of Agriculture and Rural Affairs, National Engineering Research Center for Sugarcane, College of Agriculture, Fujian Agriculture and Forestry University, Fuzhou, 350002 China; 2 State Key Laboratory of Tropical Crop Breeding, Institute of Tropical Bioscience and Biotechnology, Sanya Research Institute, Chinese Academy of Tropical Agricultural Sciences, Sanya, 572024 China

**Keywords:** NLR immune receptor, Asymmetric selection, Growth-defence trade-off, Broad-spectrum resistance

## Abstract

A recent study in *Nature* identified the rice (*Oryza sativa*) nucleotide-binding site and leucine-rich repeat (NLR) receptor XA48, which recognized the ancient bacterial effector XopG and triggered immunity by promoting degradation of the negative regulators OsVOZ1/2. Population genomics revealed an asymmetric selection, with *Xa48* retained in *indica* but lost in *japonica* due to a reproductive penalty. By stacking XA48-mediated effector-triggered immunity (ETI) with XA21-mediated pattern-triggered immunity (PTI) and introducing the compatible Os*VOZ1*^*S*^ allele, broad-spectrum bacterial blight resistance from wild rice was reconstituted. This work provides a design strategy that helps break the growth-defence trade-off.

## Main text

For decades, enhancing disease resistance in crops has often compromised yield, a trade-off shaped by natural and artificial selection. The selective dynamics reshaping immune modules during domestication remain largely unknown. Rice bacterial blight, caused by *Xanthomonas oryzae* pv. *oryzae* (*Xoo*), has resurged as a major threat (Li et al. 2025). Notably, most cloned resistance (*R*) genes (known as *Xa* genes) against this disease originate from wild relatives or represent recessive susceptibility alleles (Li et al. 2012), suggesting that resistance may have undergone loss-of-function selection during domestication. Traditional breeding has long prioritized high and stable yield. Given the marked differences between two rice (*Oryza sativa*) subspecies (*indica* and *japonica*) in disease resistance and reproductive isolation, understanding how *R* genes were evolved during domestication, especially during subspecies formation, is critical for balancing durable resistance with high yield in modern breeding.

In a landmark study published in *Nature*, Lin et al. (Lin et al. 2026) dissected this mystery by tracing the evolutionary trajectory of a rice immune module, revealing why a functional *R* gene was retained in one subspecies but purged from the other, and demonstrating how this insight can be used to rebuild resistance in a yield-compatible manner.

**Fig. 1:**
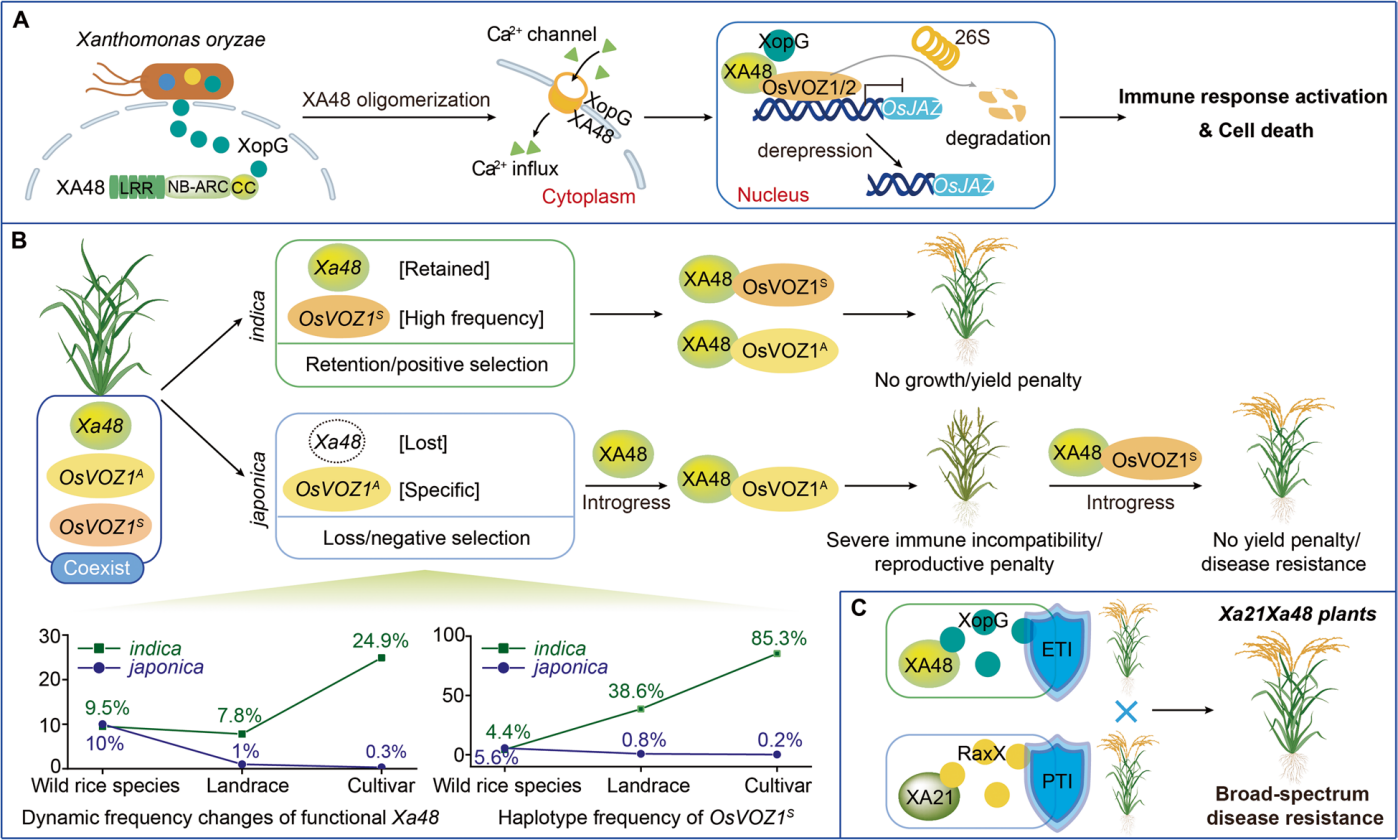
Asymmetric selection of the XA48–OsVOZ1 immune module and its rebuilding for broad-spectrum resistance. **A** XA48-mediated effector-triggered immunity (ETI). *Xanthomonas oryzae* pv. *oryzae* (*Xoo*) secretes the conserved type III effector XopG. The rice NLR receptor XA48 recognizes XopG and undergoes oligomerization to form a Ca^2+^-permeable channel at the plasma membrane, leading to Ca^2+^ influx into the cytoplasm. This triggers immune signaling that promotes the degradation of the transcriptional repressors OsVOZ1 and OsVOZ2 in the nucleus. Degradation of OsVOZ1/2 derepresses *OsJAZs*, thereby activating jasmonate-mediated immune responses and cell death. **B** Subspecies-specific selection of the XA48-OsVOZ1 module. In *indica*, both *Xa48* and the two *OsVOZ1* haplotypes (*OsVOZ1*^*A*^ and *OsVOZ1*^*S*^) coexist, enabling resistance without a yield penalty. In contrast, *japonica* has lost functional *Xa48* and retains only the *OsVOZ1*^*A*^ allele. Reintroduction of *Xa48* into *japonica* causes severe immune incompatibility and reproductive penalty. Population genomics analysis shows that the functional *Xa48* allele underwent positive selection in *indica* (increasing from landrace to cultivar) but negative selection in *japonica* (nearly lost). *OsVOZ1*^*S*^ frequency increased markedly only in *indica*. **C** Rebuilding broad-spectrum resistance by stacking PTI and ETI. Stacking of XA21-mediated pattern-triggered immunity (PTI, via recognition of RaxX) and XA48-mediated ETI (recognition of XopG) confers broad-spectrum resistance to diverse *Xoo* strains, including those that are virulent to each single gene. The *Xa21Xa48* plants have enhanced field resistance without compromising yield. This design strategy effectively integrates cell-surface and intracellular immune receptors to reconstitute wild-rice-like broad-spectrum resistance in modern rice cultivars

The authors identified a novel nucleotide-binding site and leucine-rich repeat (NLR) protein, designated XA48, in the *indica* variety Shuangkezao (SKZ) using map-based cloning and genome-wide association studies (GWAS) of 1,945 rice accessions (Lin et al. 2026). Functional validation via CRISPR-Cas9 knockout and complementation confirmed that *Xa48* confers race-specific, lifetime resistance to Northeast Asian strains of *Xoo* (Fig. 1A). Unlike many known *Xa* genes that are effective only in adult plants (Yang et al. 2022), *Xa48* functions from seedling to adult stage, highlighting its potential for breeding applications.

The cognate avirulence effector recognized by XA48 is XopG, a conserved, ancient metalloprotease effector present in diverse Gram-negative bacteria. XA48 directly binds XopG through its coiled coil domain and assembles into a calcium-permeable resistosome channel that triggers hypersensitive cell death, a hallmark of effector-triggered immunity (ETI). This aligns with findings that NLR resistosomes such as ZAR1 and Sr35 (Bi et al. 2021) function as cation channels. However, the high-resolution structure of the XA48-XopG resistosome remains undetermined, and the physiological substrate(s) of XopG in rice cells have not been identified. Full-length, functional XopG orthologs are predominantly found in Northeast Asian *Xoo* strains, suggesting that XA48-mediated resistance may be effective only against specific pathogen populations, a consideration worth bearing in mind for breeding efforts outside Northeast Asia.

A key discovery lies in the downstream signaling cascade of XA48. Using a yeast two-hybrid screen, the authors identified two vascular plant one-zinc-finger transcription factors, OsVOZ1 and its homologue OsVOZ2, both as direct interactors of XA48 (Lin et al. 2026). OsVOZ1/2 suppress jasmonate signaling and negatively regulate both pattern-triggered immunity (namely PTI, basal immunity) and XA48-mediated ETI. Upon XopG perception, the XA48-XopG complex promotes metalloprotease-dependent degradation of OsVOZ1/2 in the nucleus, leading to reduced *OsJAZ* expression and de-repression of jasmonate-mediated defense responses (Fig. 1A).

Comparing VOZ functions across pathosystems reveals an intriguing pattern. During plant immunity, the role of OsVOZ1/2 appears to be pathogen-dependent. In the case of rice blast resistance mediated by the NLR immune receptor Piz-t, both OsVOZ1 and OsVOZ2 contribute positively to ETI, and their double silencing compromises Piz-t-mediated resistance (Wang et al. 2021). In contrast, Lin and colleagues found that these same transcription factors negatively regulate basal immunity as well as XA48-mediated ETI against bacterial blight (Lin et al. 2026). This raises the possibility that VOZ transcription factors are active signaling hubs whose regulatory function is modulated by distinct NLR-effector complexes in a pathogen-specific manner. The degradation of OsVOZ1/2 depends on the presence of both XA48 and XopG, yet whether XopG directly cleaves these transcription factors or instead promotes their degradation by recruiting an endogenous rice protease or E3 ligase remains unknown (Wang et al. 2021).

The most compelling part of the study addresses the subspecies-specific asymmetric selection of this module. Population genomic analysis of over 2,451 rice accessions (including wild rice and cultivated varieties) revealed a striking pattern: functional *Xa48* alleles are found exclusively in *indica* subspecies and have been largely lost from *japonica*. During domestication and improvement, the frequency of functional *Xa48* increased from 7.8% to 24.9% in *indica*, indicating positive selection. Conversely, the allele was nearly eliminated in temperate *japonica*, declining from 1.0% to 0.3% (Fig. 1B).

Why this asymmetry? The answer lies in the *OsVOZ1* locus. *OsVOZ1* has two major haplotypes, *OsVOZ1*^*A*^ (alanine) and *OsVOZ1*^*S*^ (serine). *Indica* retains both alleles, whereas *japonica* carries almost exclusively *OsVOZ1*^*A*^. When the authors introduced *Xa48* into *japonica* varieties carrying the *OsVOZ1*^*A*^ allele, the transgenic plants showed a severe reduction in seed setting and grain yield across multiple seasons and locations. Interestingly, this yield penalty was not observed when *Xa48* was introduced into *indica* backgrounds or into *japonica* lines engineered to express the *OsVOZ1*^*S*^ allele. In other words, the XA48-OsVOZ1^A^ combination creates a genetic immune incompatibility that severely compromises reproductive development, explaining why *Xa48* was strongly selected against in *japonica* rice.

Placing this incompatibility in a broader framework reveals its similarity to hybrid necrosis or hybrid weakness, in which novel genetic combinations inadvertently activate autoimmune responses in the absence of pathogens (Calvo-Baltanás et al. 2021). In rice, similar incompatibilities have been mapped to two-locus interactions, including the *Hwc1*/*Hwc2* locus pair that causes F_1_ hybrid weakness (Chen et al. 2014). Molecular characterization revealed that this weakness involves constitutive activation of autoimmunity, including the accumulation of salicylic acid and jasmonic acid and the upregulation of pathogenesis-related genes (*PRs*) (Chen et al. 2014). That is, the XA48-OsVOZ1^A^ incompatibility adds to a growing list of cases where NLRs, when combined with incompatible genetic backgrounds, compromise reproductive fitness. This framework explains why *Xa48* was selectively purged from *japonica* during domestication: the fitness cost of maintaining this *R* gene outweighed its benefit in regions where *XopG*-positive strains are less prevalent.

Leveraging these insights, the authors (Lin et al. 2026) engineered *japonica* lines to co-express *Xa48* (mediating ETI) and the well-known pattern-recognition receptor gene *Xa21*, which mediates PTI by recognizing the conserved RaxX peptide, providing a complementary recognition spectrum (Song et al. 1995). The resulting *Xa21Xa48* plants were resistant to 30 *Xoo* strains, including those virulent to either gene alone (Fig. 1C). Furthermore, by reconstituting the XA48-OsVOZ1^S^ immune module into *japonica*, which combines a functional NLR with a reproductively compatible VOZ allele, they restored resistance without yield penalty. These findings support a revised model in which PTI potentiation is indispensable for ETI during bacterial infection (Yuan et al. 2021), conceptually uniting two immune signaling cascades and explaining how their combination reconstitutes the broad-spectrum resistance observed in wild rice.

Lin and colleagues (Lin et al. 2026) provide a new evolutionary paradigm: the fate of an *R* gene is determined not only by its efficacy against pathogens but also by its genetic compatibility with host reproductive and developmental programs. A key future question is whether similar asymmetric selection of NLR transcription factor modules occurs in other crops, requiring large-scale pan-genomic analyses.

Collectively, Lin et al. (Lin et al. 2026) offer a strategy for rebuilding resistance in modern crops without compromising yield by dissecting the molecular basis of genetic incompatibility. This work provides a framework for sustainable agriculture in the face of evolving pathogens, showing that the most promising path forward is not to rely on single genes but to pursue the deliberate, multi-layered engineering of entire immune networks shaped by both natural and artificial selection.

## Data Availability

Not applicable.

## Abbreviations

ETI: Effector-triggered immunity
GWAS: Genome-wide association studies
NLR: Nucleotide-binding site and leucine-rich-repeat receptor
PRs: Pathogenesis-related genes
PTI: Pattern-triggered immunity
*R* genes: Resistance genes
SKZ: Shuangkezao
*Xoo*: *Xanthomonas oryzae* Pv. *oryzae*
